# Development and Field Test of Integrated Electronics Piezoelectric Accelerometer Based on Lead-Free Piezoelectric Ceramic for Centrifugal Pump Monitoring

**DOI:** 10.3390/s24196436

**Published:** 2024-10-04

**Authors:** Byung-Hoon Kim, Dae-Sic Jang, Jeong-Han Lee, Min-Ku Lee, Gyoung-Ja Lee

**Affiliations:** Nuclear System Integrity Sensing & Diagnosis Division, Korea Atomic Energy Research Institute, Daejeon 34057, Republic of Korea; bhkim@kaeri.re.kr (B.-H.K.); bigplant@kaeri.re.kr (D.-S.J.); jhleeyo@kaeri.re.kr (J.-H.L.)

**Keywords:** lead-free piezoelectrics, (K,Na)NbO_3_-based ceramics, accelerometer, field test, centrifugal pump

## Abstract

In this study, an Integrated Electronics Piezoelectric (IEPE)-type accelerometer based on an environmentally friendly lead-free piezoceramic was fabricated, and its field applicability was verified using a cooling pump owned by the Korea Atomic Energy Research Institute (KAERI). As an environmentally friendly piezoelectric material, 0.96(K,Na)NbO_3_-0.03(Bi,Na,K,Li)ZrO_3_-0.01BiScO_3_ (0.96KNN-0.03BNKLZ-0.01BS) piezoceramic with an optimized piezoelectric charge constant (*d*_33_) was introduced. It was manufactured in a ring shape using a solid-state reaction method for application to a compression mode accelerometer. The fabricated ceramic ring has a high piezoelectric constant *d*_33_ of ~373 pC/N and a Curie temperature *T*_C_ of ~330 °C. It was found that the electrical and physical characteristics of the 0.96KNN-0.03BNKLZ-0.01BS piezoceramic were comparable to those of a Pb(Zr,Ti)O_3_ (PZT) ring ceramic. As a result of a vibration test of the IEPE accelerometer fabricated using the lead-free piezoelectric ceramic, the resonant frequency *f*_r_ = 20.0 kHz and voltage sensitivity *S*_v_ = 101.1 mV/g were confirmed. The fabricated IEPE accelerometer sensor showed an excellent performance equivalent to or superior to that of a commercial IEPE accelerometer sensor based on PZT for general industrial use. A field test was carried out to verify the applicability of the fabricated sensor in an actual industrial environment. The test was conducted by simultaneously installing the developed sensor and a commercial PZT-based sensor in the ball bearing housing location of a centrifugal pump. The centrifugal pump was operated at 1180 RPM, and the generated vibration signals were collected and analyzed. The test results confirmed that the developed eco-friendly lead-free sensor has comparable vibration measurement capability to that of commercial PZT-based sensors.

## 1. Introduction

Piezoelectric accelerometers are key sensors for monitoring and diagnosing the status of major devices and structures in various industrial fields, such as nuclear power plants, chemical plants, automobiles, aerospace, and construction [1,2]. In particular, they play a key role in reducing the maintenance costs and enhancing the safety and reliability of equipment by detecting early wear or failure of major devices and precisely diagnosing their status to prevent accidents in advance. Piezoelectric accelerometers are typically used as charge-type without built-in electronic circuits and IEPE-type with built-in electronic circuits. Charge-type accelerometers are used at high temperatures above 200 °C because they do not have electronic circuits, but they have relatively low performance and electrical signal noise problems. Therefore, in mild environments below 200 °C, IEPE-type accelerometers with relatively high performance and reliability are mostly used [3]. Traditionally, these sensors have been manufactured using PZT piezoelectric ceramics containing lead as the sensing material; however, the use of hazardous substances such as lead raises environmental concerns [4,5]. Their use has been restricted for several years according to the international environmental regulation RoHS (Restriction of Hazardous Substances), however, their regulation is still being extended because sensors based on suitable eco-friendly alternative materials have not been commercialized yet [5,6,7,8].

Lead-free materials generally have lower piezoelectric properties, and thus research is being conducted to enhance their performance by controlling the phase structure and microstructure through doping or process improvements. It has been reported that BaTiO_3_ (BT)-based piezoelectric ceramics afford improved piezoelectricity through A-site, B-site, and A/B-site co-doping. Dopants such as A(Cu_1/3_Nb_2/3_)O_3_ (A=Ca^2+^, Sr^2+^, Ba^2+^) [9], Li^+^ [10], and Sn^4+^/Y^3+^ [11] have been used, resulting in an increased piezoelectric constant (*d*_33_) of approximately 454 pC/N. However, the Curie temperature (*T*_C_) remains low, at 81 °C, limiting the material’s usable temperature range [11]. Meanwhile, Bi_1/2_Na_1/2_TiO_3_ (BNT)-based piezoelectric ceramics have shown improved piezoelectric properties through the formation of binary and ternary systems using (Bi_0.5_K_0.5_)TiO_3_, (Bi_0.5_Li_0.5_)TiO_3_, and BaTiO_3_ [12,13,14,15]. While the piezoelectric constant was improved to approximately 231 pC/N, a depolarization temperature (*T*_d_) of roughly 125°C was observed [15]. Despite improvements in the piezoelectric properties of both BT-based and BNT-based ceramics, their lower *T*_C_ and *T*_d_ are barriers to replacing PZT-based piezoelectric ceramics.

Meanwhile, (K,Na)NbO_3_ (KNN)-based piezoelectric ceramics have attracted significant attention, as they exhibit a high Curie temperature and a piezoelectric constant comparable to that of PZT-based ceramics. Research to enhance the performance of KNN-based piezoelectric ceramics by using dopants such as Li^+^ [16,17,18], Ta^5+^ [19,20], Sb^5+^ [21,22], and ABO_3_-type perovskite compounds [23,24,25,26,27] is actively underway. Among the dopants, Sb^5+^ has been extensively studied for its ability to effectively improve piezoelectric properties. However, significantly lowering the Curie temperature has been a drawback, and research is ongoing to overcome this limitation [27]. Studies have reported achieving a Curie temperature of above 400 °C and a piezoelectric constant greater than 200 pC/N through Li^+^ and Ta^5+^ doping [16,17,18,19,20]. Additionally, the formation of binary and ternary systems using LiNbO_3_, BiScO_3_, and (Bi,M)ZrO_3_ (M = Na, Li, K, Ag) has led to the development of KNN-based piezoceramics with a Curie temperature of above 300 °C and a piezoelectric constant exceeding 300 pC/N [21,22,23,24,25,26,27].

In a previous study, our research team investigated the changes in the phase structure, microstructure, and electrical properties according to changes in x and y for (1-x-y)KNN-xBNKLZ-yBS ternary ceramics. We reported that the maximum piezoelectric response was observed at a specific rhombohedral–tetragonal (R-T) phase boundary. As a result, an R-T phase boundary consisting of approximately 15% R phase and 85% T phase was observed at 0.96KNN-0.03BNKLZ-0.01BS, and a maximum piezoelectric constant *d*_33_ of ~370 pC/N and Curie temperature *T*_C_ of ~332 °C were confirmed [24]. The present paper is focused on the development of a voltage-type accelerometer by incorporating this optimal piezoelectric ceramic (0.96KNN-0.03BNKLZ-0.01BS) into a self-developed IEPE circuit, as well as evaluating the potential of the developed sensor for field application through excitation and field tests.

The compressive piezoelectric accelerometer consists of parts such as a head (mass), screw, piezoceramic rings, tail, insulating layer, and base, and several piezoceramic rings are electrically connected in parallel and mechanically connected in a vertical structure. The piezoelectric ceramics are compressively inserted between the mass and the tail using a screw. In a previous study, four prototypes of charge-type accelerometer were designed through the numerical optimization of the design variables of each component, and the performance of the accelerometer prototypes manufactured with 0.96KNN-0.03BNKLZ-0.01BS ceramic rings was investigated theoretically and experimentally. As a result, the developed accelerometer prototype had a high level of charge sensitivity *S*_q_ (55.1~223.8 pC/g) and resonant frequency *f*_r_ (14.1~28.4 kHz), which showed almost the same performance as the PZT-based accelerometer [28].

In this study, a voltage-type accelerometer sensor based on lead-free piezoelectric ceramics was manufactured by introducing the self-developed IEPE circuit and housing to the charge-type accelerometer prototype. In general, IEPE accelerometers can be manufactured with a resonant frequency of up to 180 kHz depending on the design, but accelerometers with a resonant frequency of 20 to 30 kHz are widely used for a relatively higher output. Therefore, the design of the lead-free accelerometer prototype was selected as a design with a resonant frequency of approximately 20 kHz, and 0.96KNN-0.03BNKLZ-0.01BS was used as the piezoelectric sensing material. The self-made IEPE circuit was applied to the accelerometer prototype to control the sensor signal, enabling more precise and reliable measurements. In order to verify the performance of the manufactured IEPE accelerometer sensor, the voltage sensitivity and frequency characteristics were evaluated by using a vibration system. In addition, a field test was performed using a cooling pump owned by KAERI. The performance of the lead-free KNN-based sensor was compared to that of a PZT-based commercial sensor used in general industrial sites under the same conditions. Based on these results, we aim to demonstrate the industrial applicability of the developed lead-free accelerometer.

## 2. Materials and Methods

### 2.1. Preparation and Characterization of Ring-Shaped Piezoceramic Samples

**Figure 1:**
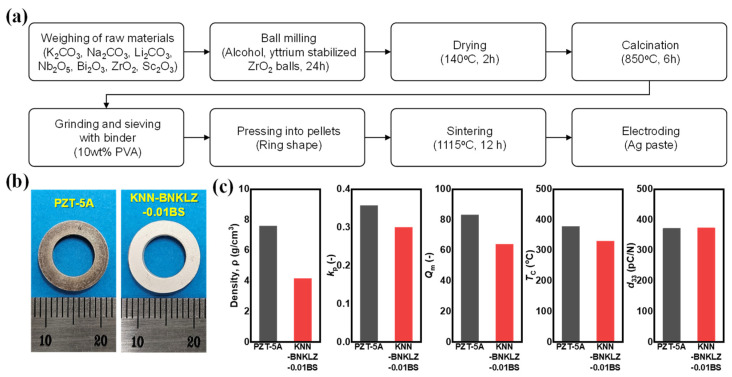
(**a**) Flow chart of piezoceramic fabrication procedure. (**b**) Actual photo of PZT-5A and 0.96KNN-0.03BNKLZ-0.01BS ceramic rings. (**c**) Density, *k*_p_, *Q*_m_, *T*_C_, and *d*_33_ values.

Polycrystalline 0.96KNN-0.03BNKLZ-0.01BS ceramic rings, which are essential for sensor fabrication, were prepared through solid-state synthesis, as shown in Figure 1a. The raw material powders used were K_2_CO_3_ (≥99.5%, FUJIFILM Wako Pure Chemical Corporation, Osaka, Japan), Na_2_CO_3_ (≥99.5%, 10 μm, Sigma-Aldrich, Burlington, NJ, USA), Li_2_CO_3_ (99.997%, 20 μm, Sigma-Aldrich, Burlington, NJ, USA), Nb_2_O_5_ (99.9%, DAEJUNG Chemical, Gyeonggi-do, Republic of Korea), Bi_2_O_3_ (99.9%, 10 μm, Sigma-Aldrich, Burlington, NJ, USA), ZrO_2_ (99.0%, 5 μm, Sigma-Aldrich, Burlington, NJ, USA), and Sc_2_O_3_ (≥99.9%, 10 μm, Sigma-Aldrich, Burlington, NJ, USA). Stoichiometric power mixtures of these raw material powders were homogenized through a process of ball milling in alcohol, dried, and calcined at 850 °C for 6 h. The calcined powder was mixed with polyvinyl alcohol (PVA) binder, and the mixed powder was compressed into a ring shape with an outer diameter of 15 mm and an inner diameter of 9 mm. The molded body was heat-treated at 650 °C for 30 min to burn off the internal binder and then sintered at 1115 °C for 12 h. Actual photographs of the 0.96KNN-0.03BNKLZ-0.01BS piezoelectric ceramic ring and the PZT-5A ceramic ring used for characteristic comparison are shown in Figure 1b. The outer diameter of the PZT-5A ceramic ring was 12.62 mm, the inner diameter was 7.52 mm, and the thickness was 2.57 mm, whereas the KNN ceramic ring had an outer diameter of 12.40 mm, an inner diameter of 7.46 mm, and a thickness of 2.56 mm, showing almost similar dimensions and shapes between the two ceramics.

Density measurements of the ceramic rings were precisely performed using the Archimedes method, which is based on the weight change that occurs when the ceramic ring is completely immersed in water. The density values obtained through these measurements provide important information about the internal structure and porosity of the ceramic. Electrodes were formed on the upper and lower surfaces of the ceramic ring to measure the electrical, ferroelectric, and piezoelectric properties. The silver paste was coated on the upper and lower surfaces of the sample and then heat-treated at 650 °C for 10 min. During the poling process, a DC electric field of 20 kV/cm was applied for 1 min at room temperature to form a permanent electric field inside of the ceramic, which is essential for activating the piezoelectric properties of the ceramic ring.

The prepared samples were heated at 5 °C/min from room temperature to 500 °C using an impedance analyzer (SI 1260; Solartron, Farnborough, UK) to measure the dielectric constant (*ε*_r_), loss factor (*tan*δ), and Curie temperature (*T*_C_). The static piezoelectric coefficient d_33_ of the samples was measured at room temperature using a piezo-*d*_33_ meter (ZJ-6B; IACAS, Beijing, China). In addition, the antiresonant frequency (*f*_a_) and resonant frequency (*f*_r_) of the ceramics were confirmed with an impedance frequency sweep using an impedance analyzer (HP 4294A; Agilent, Santa Clara, CA, USA), and the electromechanical coupling factor (*k*_p_) was determined from these data. Additionally, the mechanical quality factor (*Q*_m_) was determined through the impedance (*Z*_r_) at the resonant frequency and capacitance at 1 kHz.

### 2.2. IEPE Accelerometer Fabrication and Basic Performance Verification

**Figure 2:**
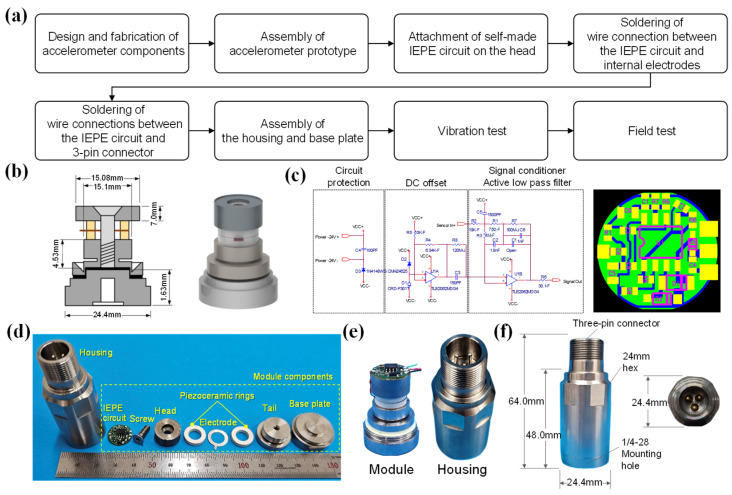
(**a**) Flow chart of manufacturing and testing process of an IEPE accelerometer. (**b**) Accelerometer prototype (**left**) part dimensions (head outer diameter, head height, tail outer diameter (O.D.), tail height, base outer diameter (O.D.), base height) and (**right**) three-dimensional images. (**c**) Designed IEPE circuit diagram (**left**) and Gerber file for PCB fabrication (**right**). (**d**) Accelerometer sensor components and 0.96KNN-0.03BNKLZ-0.01BS ceramic ring. (**e**) Accelerometer prototype with IEPE circuit attached (**left**) and 3PIN housing (**right**). (**f**) Fabricated IEPE-type accelerometer sensor and external dimensions.

An IEPE accelerometer sensor was fabricated using a 0.96KNN-0.03BNKLZ-0.01BS ceramic ring, components of a charge-type accelerometer prototype based on the compression mode operation principle, housing that fits it, and a self-made IEPE circuit. The accelerometer consists of a total of nine components, and detailed information about each component is summarized in Table 1. The manufacturing and testing process of an IEPE accelerometer using the prepared components is shown in the Figure 2a. Epoxy was applied between the tail and the base plate to connect the two parts and serve as an insulator. The epoxy applied between the tail and the base plate was heat-treated at 140 °C for 1 h to maintain a constant thickness and strength. The prepared accelerometer prototype components were assembled using a torque wrench at a torque of 1.5 N∙m. The self-made IEPE circuit board was attached to the upper surface of the head of the assembled accelerometer prototype using conductive silver paste. The silver paste was cured at 110 °C for 1 h, and this condition was set considering the operating temperature of the circuit and the curing condition of the silver paste. In order to transmit the electrical signal of the accelerometer prototype to the IEPE circuit, the internal electrodes and the IEPE circuit were connected with soldering wires. Finally, the 3-pin connector and the IEPE circuit were connected, and the housing was assembled to the base plate to complete the accelerometer.

In order to precisely evaluate the performance of the fabricated accelerometer, this study designed and built a highly precise excitation system that can provide vibrations of up to 40 g in the range of 5 Hz to 50 kHz under room temperature conditions. This evaluation system consists of the following devices: a vibration generator (SE-09, SPEKTRA GmbH, Dresden, Germany) to generate vibrations, a power amplifier (PA 14-500, SPEKTRA GmbH, Dresden, Germany) to amplify the vibration signals, a function generator to generate various vibration signals, a DAQ system (m+p VibPilot, m+p International, Hannover, Germany) to collect the vibration signals generated from the sensor by excitation, software to analyze the signals received from the DAQ (Acquisition software m+p VibControl Revision 2.15, m+p International, Hannover, Germany), an internal reference standard accelerometer (BN-09, sensitivity = 10 mV/g, resonance frequency = 70 kHz) for sensor calibration, a power supply (LRS-50-24, Mean Well, new Taipei, Taiwan), and a signal conditioner (133, Endevco, Irvine, CA, USA) to supply power to the circuit.

### 2.3. Field Test of IEPE Accelerometer (Data Acquisition and Signal Analysis)

A field test was conducted to verify the field applicability of the developed sensor by comparing its signal detection capabilities with those of a commercial sensor in an environment containing various noises. First, both the developed sensor and the commercial sensor were attached to the ball bearing housing of a cooling pump owned by the KAERI, and their signal detection capabilities were analyzed by comparing the statistical characteristics of the vibration signals in the time domain. Subsequently, the time-domain signals were converted into the frequency domain, and the sensors’ abilities to detect the frequency components related to typical defects of ball-bearing-type centrifugal pumps were compared to further validate the developed sensor’s field applicability.

Acquisition hardware (Agilent VXI, Keysight Technologies, Santa Rosa, CA, USA) and acquisition software (I-deas TDAS 63.0.0.6, B&K, Dærum, Denmark) were used for signal collection and analysis. The signal collection and processing conditions were set as follows: Sampling rate: 32,768 Hz, Block size: 65,356, df: 0.5 Hz, Window option: Hanning, Overlap: 50%, Average No.: 200, and Frequency range: 10~1000 Hz. The information about the test pump is as follows: Pump type: centrifugal pump with ball bearing, Revolution: 1180 RPM, Brake horsepower (BHP): 260.8 kW, Head: 40 m, and Capacity: 30 m^3^/min.

## 3. Results

The density and piezoelectric properties of the 0.96KNN-0.03BNKLZ-0.01BS and PZT-5A ring ceramics are shown in Figure 1c. The density of the 0.96KNN-0.03BNKLZ-0.01BS ceramic ring was confirmed to be 4.15 g/cm^3^, which is approximately 92% of the theoretical density (ref. Pure KNN 4.51 g/cm^3^). The electromechanical coupling coefficient (*k*_p_) and the mechanical quality factor (*Q*_m_) were determined to be 0.30 and 64, respectively, through the impedance analysis. The phase transition temperature (*T*_C_) and piezoelectric constant (*d*_33_) were confirmed to be 330 °C and 373 pC/N, respectively. *T*_C_ is related to the operating temperature, and *d*_33_ is proportional to the charge sensitivity of the compressive accelerometer sensor [28,29,30]. These two important characteristics were almost equivalent to commercial PZT-5A [8]. Therefore, 0.96KNN-0.03BNKLZ-0.01BS piezoelectric ceramics showed the possibility of sufficiently replacing the PZT-5A used in compression-type accelerometers.

The dimensions of each component of the accelerometer prototype and its three-dimensional image are shown in Figure 2b [28]. According to this design, the accelerometer prototype components, IEPE circuit, and housing were manufactured. The IEPE circuit was designed as a charge mode and a three-wire output, and an operational amplifier (Op Amp), capacitors, resistors, current regulatory diodes, Zener diodes, and diodes were used as the circuit components [3]. The manufactured circuit was designed to consist of circuit protection, DC offset, a signal conditioner, and an active low-pass filter using the above components. Circuit protection is configured using diodes and capacitors; DC offset is configured using op amps; and Zener diodes, regulative diodes, capacitors, resistors, signal conditioners, and active low-pass filters are configured using op amps, capacitors, and resistors. Circuit protection prevents reverse current flow and prevents high voltage and overcurrent, DC offset adjusts the DC level, signal conditioners amplify and filter, and active low-pass filters pass low-frequency signals and remove high-frequency signals. The frequency response characteristics and voltage sensitivity of the accelerometer were optimized by adjusting the electrostatic capacitance of the capacitor and the resistance value of the resistor. Since the piezoelectric accelerometer uses a region that has flat output characteristics below the mounted resonance frequency, the frequency range with a voltage sensitivity of about 100 mV/g was adjusted to be the maximum. The optimized circuit diagram and the drawing file (Gerber file) for manufacturing the printed circuit board (PCB) are shown in Figure 2c.

An actual photo of the IEPE circuit, housing, and accelerometer prototype components is shown in Figure 2d. The piezoceramic rings, which are responsible for the core function of the accelerometer sensor, were made of two 0.96KNN-0.03BNKLZ-0.01BS piezoceramic rings, as shown in Figure 1. The housing, screw, tail, and base plate were made of stainless steel, and the electrodes and wires, which transmit electrical signals, were made of Inconel and Cu wire, respectively. The head component, which determines the sensitivity of the accelerometer sensor, was designed to realize high sensitivity by utilizing high-density tungsten. The left side of Figure 2e is a sensor module manufactured by assembling the accelerometer sensor components, excluding the housing, and the right side is a photo of the housing manufactured with a three-pin (3PIN) connector according to the manufactured IEPE circuit. An IEPE accelerometer with an outer diameter of 64.0 mm and a diameter of 24.4 mm was manufactured, as shown in Figure 2f. The 1/4-28 mounting hole on the bottom of the base was designed to be attached to a measuring device or a test structure using a mounting stud, and the performance was evaluated after electrically connecting the 3PIN connector of the accelerometer using a dedicated cable.

**Figure 3:**
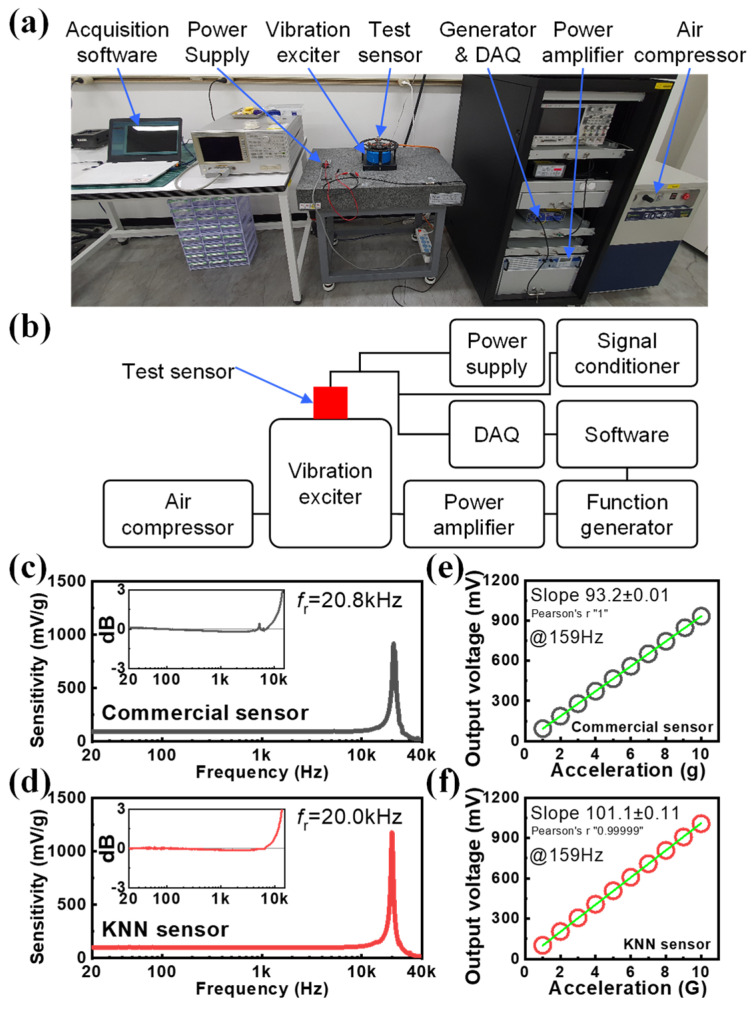
(**a**) The photo and (**b**) block diagram of excitation system for vibration test. Frequency response profiles of (**c**) commercial PZT-based sensor and (**d**) KNN-based sensor measured through vibration experiments. Voltage vs. acceleration characteristics (test frequency = 159 Hz) of (**e**) commercial PZT-based sensor and (**f**) KNN-based sensor.

Figure 3a shows an excitation system for a precise vibration experiment, and the voltage sensitivity and frequency response characteristics of the IEPE accelerometer were evaluated. This excitation system was configured as shown in Figure 3b, and the vibration experiment was performed in the range of 20 Hz to 40 kHz and 1 to 10 g (g, gravitational acceleration = 9.8 m/s^2^). Vibration was applied to the sensor using a vibration exciter, power amplifier, and function generator, and the applied acceleration was determined using the electrical signal generated by the reference accelerometer installed inside of the equipment. The KNN-based sensor developed in this study does not require a signal conditioner, because the signal conditioner function is built into the internal IEPE circuit. However, since a stable power supply is essential to drive the sensor circuit, −24 V was supplied to the end of the cable connected to the sensor using a separate power supply. The electrical signal generated by the applied vibration was collected by the DAQ, and the signal was analyzed using dedicated software.

In order to objectively analyze the performance of the KNN-based sensor, a commercial PZT-based sensor (SKF, CMSS 2106, 100 mV/g sensitivity, 20 kHz mounted resonance frequency), which is widely used in general industries, was selected and evaluated under the same conditions for comparative analysis. Since this commercial PZT-based sensor consists of a two-pin (2PIN) connector, the circuit power was driven through a signal conditioner instead of a power supply to conduct the evaluation.

Figure 3c,d show the frequency response profiles of the PZT-based commercial sensor and the KNN-based sensor. The resonant frequency of both sensors was observed at approximately 20 kHz, and the additionally provided inset figure confirmed the frequency flatness characteristics of both sensors from approximately 20 Hz to 10 kHz with a ±3 dB criterion. This indicates that both sensors provide stable performance, even in the high-frequency band. Figure 3e,f show the voltage acceleration characteristics, measured in the range of 1 g to 10 g at 159 Hz. From the measurement results, we can see that both sensors showed perfect linear response characteristics with Pearson’s r values close to 1. The measured voltage sensitivity was 93.2 mV/g for the commercial PZT-based sensor, while it was 101.1 mV/g for the KNN-based sensor. Both sensors were designed to be 100 mV/g, and the commercial PZT-based sensor presented a specification of 100 mV/g. The KNN-based sensor was confirmed to have an error within approximately ±2% of the specifications, and the commercial PZT-based sensor was confirmed to have an error within approximately ±7%. Therefore, the vibration test results confirmed that the frequency and sensitivity characteristics of the KNN-based sensor were superior to the Pb-based commercial sensor.

To confirm the applicability of the developed KNN accelerometer in an actual industrial environment, a field test was conducted on a rotating machine. The target pump for the test was a ball-bearing-type centrifugal pump, which is a pump commonly used to circulate cooling water in an industrial environment. The accelerometer sensor is installed in a ball-bearing-type centrifugal pump in many actual industrial sites and is used to diagnose the status of the pump [31,32,33]. In this study, the developed eco-friendly lead-free sensor and the commercial PZT-based sensor were attached to the test target pump, and the detection performance of the two sensors for vibrations generated during pump operation was compared and evaluated.

**Figure 4:**
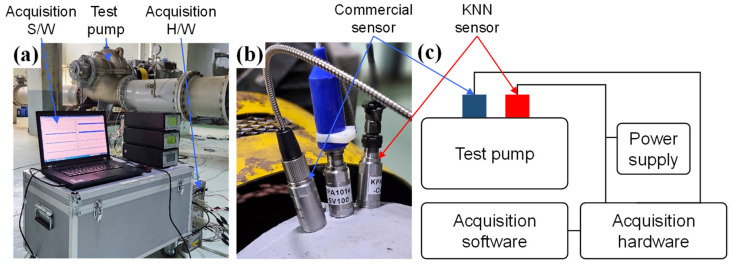
(**a**) Photo of centrifugal pump field test. (**b**) Photograph of the commercial PZT-based sensor and KNN-based sensor attached to the bearing housing. (**c**) Field test block diagram.

Figure 4a,b show the test target pump, the installed measurement system, and the attached sensors for pump vibration measurement. In order to install the commercial PZT-based sensor and KNN sensor on the pump, the adhesive mounting base was attached using a room-temperature curing adhesive, and the two sensors were fastened to the 1/4-28 stud of the adhesive mounting base. In order to accurately transmit the pump vibration to the sensor, the sensor should be fastened with an appropriate torque using a dedicated mounting stud after processing a tap on the pump surface. However, the adhesive mounting base method was used instead of the tap processing method to prevent damage to the pump in operation on site. It is known that the mounted resonance frequency of the sensor is relatively reduced when using the adhesive mounting base compared to the tap processing method. Therefore, when analyzing the vibration signal data generated through the field test, a signal below 1 kHz was used, considering this frequency characteristic change. The mounting location of the sensor in the centrifugal pump can be horizontal, vertical, or axial to the bearing, according to the ISO 20816-1, ISO 10816-7 standard [34,35]. In this test, the sensor was installed in the vertical direction to the bearing, which has a high vibration sensitivity of the rolling bearing. In addition, the rotating machine has a Drive End (DE) position, where the driving force is transmitted, and the position with high vibration energy is known as the DE position. Therefore, in order to well reflect the vibration characteristics of the pump, the sensor was attached vertically to the bearing housing of the DE position of the target pump. The sensors were connected to the DAQ (data acquisition) system hardware to collect the vibration signal, and the collected signal was stored and analyzed using DAQ system software (I-deas TDAS 63.0.0.6, B&K, Dærum, Denmark). The KNN-based sensor was supplied with power using the voltage supply device, and the commercial PZT-based sensor was supplied with power using the DAQ system hardware’s own power supply.

**Figure 5:**
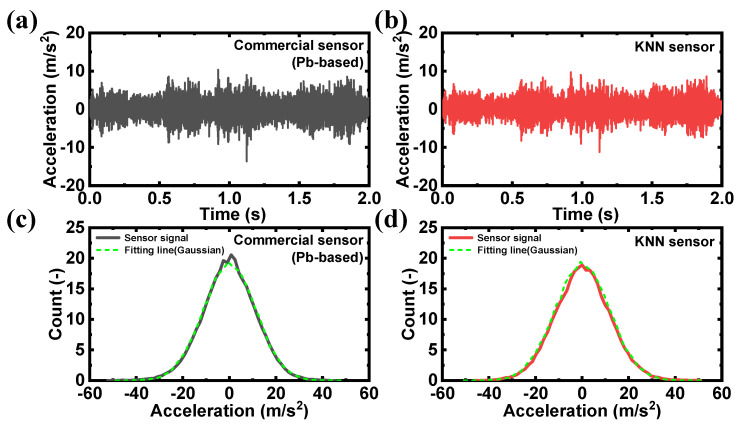
(**a**) Acceleration signals over time of the commercial PZT-based sensor and (**b**) KNN-based sensor. (**c**) Histogram and Gaussian fitting results for the average amplitude values measured with the commercial PZT-based sensor and (**d**) KNN-based sensor.

The target pump for the test was operated at 1180 RPM, and vibration signal collection was started after the pump stabilized for sufficient time after operation. Figure 5a,b show the acceleration signals collected over time from the commercial PZT-based sensor and the KNN-based sensor, respectively. Since the target pump was operated at the rated speed and load, the vibration signal of the pump measured with the sensor is known to follow Gaussian distribution, which is a general signal characteristic of a stationary random vibration signal [36,37,38]. Therefore, if the sensor signal characteristics used in this test are in a steady state, the time signal characteristics of the measured vibration signal should follow Gaussian distribution. To verify this, a histogram of the measured time signals was calculated, and the average (overlap 50%) was performed 200 times to reduce the random noise. Figure 5c,d present histograms of the time signals measured with the commercial PZT-based sensor and the KNN sensor, along with the results of Gaussian fitting using the mean and variance of the vibration signal. It was confirmed that the histograms from both sensors followed Gaussian distribution. To provide a clearer comparison, the similarity between the histogram and the Gaussian fit was measured using Jensen–Shannon divergence (JSD) [39,40], a quantitative metric that evaluates the similarity of probability distributions. Using Kullback-Leibler divergence (KLD) in Equation (1), the JSD is expressed as shown in Equation (2). The JSD value ranges from 0 to ln(2), with values closer to 0 indicating higher similarity between two probability distributions. The JSD value for the commercial PZT-based sensor’s histogram and Gaussian fit was 0.012, and that of the KNN-based sensor was 0.005, confirming that both sensors have high similarity in the histogram and Gaussian fitting. Thus, the KNN-based sensor developed in this study demonstrates signal characteristics comparable to those of the commercial PZT-based sensor.

(1) $$KLD\left(\left.x\right\|y\right)=\sum x_{i}\ln\left(\frac{x_{i}}{y_{i}}\right),\;x,\;y:\;random\;variables,\;i=1,2,3,\;\ldots ,\;n$$

(2) $$JSD\left(x,y\right)=\frac{KLD\left(\left.x\right\|M\right)+KLD\left(\left.y\right\|M\right)}{2},\;M=\frac{x+y}{2}$$

**Figure 6:**
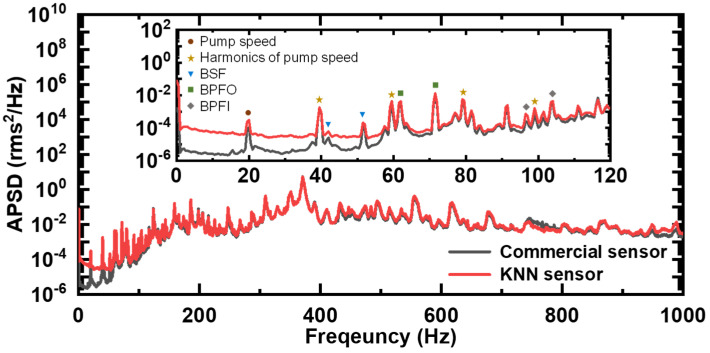
Frequency spectrum for the measured signals from the commercial PZT-based sensor and KNN-based sensor.

In order to analyze the frequency characteristics of the vibration signal, Fast Fourier Transform (FFT) was performed to convert the vibration signal of the pump into the frequency domain, and the frequency analysis was performed in the 10 to 1000 Hz band range, according to the standard of ISO 10816-7 [35]. Figure 6 shows the results of calculating the Auto-Power Spectral Density (APSD) after performing FFT on the signals generated with the commercial PZT-based sensor and the KNN sensor. The frequency spectrum confirmed that the peaks shown in the two sensor signals were similar, and the observed peaks were compared and analyzed in relation to the peak frequency calculated using the rotational speed, ball diameter, number of balls, pitch diameter, and contact angle of the ball-bearing-type centrifugal pump.

The test pump’s rotational speed was 1180 RPM, and the corresponding peak frequency was observed at 20 Hz, as shown in Figure 6. Also, peak frequencies of 39.5 Hz, 59.5 Hz, 79 Hz, and 99 Hz are the 2nd to 5th harmonic components of the pump rotational frequency, which were measured using both sensors. The locations of these peaks are indicated by symbols (circle, star) in the inset of Figure 6, and the observed peaks were confirmed at the same locations with both sensors. These peaks are commonly used to diagnose typical pump defects, such as mass unbalance, misalignment, bent shaft, eccentric rotor, rotor rub, and mechanical looseness [41].

The frequency components used for ball bearing defect diagnosis include BSF (Ball Spin Frequency), BPFO (Ball Pass Frequency Outer race), BPFI (Ball Pass Frequency Inner race), and FTF (Fundamental Train Frequency). BSF is a frequency component related to ball defects, and, since the inner and outer rings contact each other at the same time when a ball defect occurs, the 2 × BSF component is monitored as the main frequency component. In addition, when a defect occurs in the outer ring, the BPFO component becomes relatively larger, and, when a defect occurs in the inner ring, the BPFI component becomes relatively larger. When a defect occurs in the ball bearing cage, a relatively low frequency component, FTF, occurs below 10 Hz, but it was not observed in this test. The calculation formula for the frequency components corresponding to this ball bearing defect diagnosis is shown below [41,42].

(3) $$f_{BSF}=\frac{f_{r}N_{B}(1+\frac{D_{b}}{D_{p}}cos\theta )}{2}$$

(4) $$f_{BPFO}=\frac{f_{r}N_{B}(1-\frac{D_{b}}{D_{p}}cos\theta )}{2}$$

(5) $$f_{BPFI}=\frac{f_{r}D_{p}(1-2(\frac{D_{b}}{D_{p}}cos\theta ))}{2D_{B}}$$

(6) $$f_{FTF}=\frac{f_{r}(1-\frac{D_{b}}{D_{p}}cos\theta )}{2}$$

Here, *f*_r_, *N*_B_, *D*_b_, *D*_p_, and *θ* are the pump rotation speed, the number of ball bearings, the ball diameter, the pitch diameter, and the contact angle, respectivley. The results calculated using the above formula and the actual measurement results obtained in this test are shown in Table 2, and each peak is indicated by symbols (down triangle, square, and diamond) in the inset of Figure 6. Both the commercial PZT-based sensor and the KNN-based sensor showed vibration peaks at the same frequencies, and the error between the calculated frequency results and the actual results was confirmed to be within approximately ±5%.

Pump rotation frequency and its harmonics, as well as the ball bearing frequency components, are widely known as essential elements used to diagnose the failure of ball-bearing-type pumps [41,42]. As a result of analyzing the signals generated using commercial PZT-based sensors and KNN-based sensors, the existence of frequency components essential for the condition monitoring and fault diagnosis of pumps and ball bearings was confirmed. These results confirm that the developed eco-friendly accelerometer has a similar performance and reliability to commercial PZT-based sensors and show that it can be used along with commercial PZT-based sensors for condition monitoring and fault diagnosis of pumps in actual industrial fields. Furthermore, if the developed sensor is evaluated in other field tests, such as structural monitoring, vibration and fatigue evaluation of aircraft engines, and automobile acceleration evaluation, in addition to centrifugal pump vibration monitoring, it will be able to be used in more industrial fields.

## 4. Conclusions

An IEPE accelerometer sensor based on lead-free ceramics was fabricated, and the performance of the fabricated sensor and its applicability in an actual machine equipment operation environment were confirmed. The core material of the accelerometer sensor, a piezoelectric ceramic, was fabricated with a composition of 0.96KNN-0.03BNKLZ-0.01BS using a solid-state reaction method, and the piezoelectric constant (*d*_33_) and phase transition temperature (*T*_C_), which are related to the sensitivity and operating temperature of the sensor, were confirmed to be 373 pC/N and 330 °C, respectively. The IEPE accelerometer sensor was assembled and fabricated using two 0.96KNN-0.03BNKLZ-0.01BS piezoelectric ceramic rings and housing, a screw, tail, base plate, electrode, and charge mode IEPE circuit. By adjusting the capacitance and resistance of the circuit, the voltage sensitivity and frequency response characteristics were optimized, and an eco-friendly IEPE accelerometer sensor with a voltage sensitivity *S*_v_ = 101.1 mV/g, a resonant frequency *f*_r_ = 20.0 kHz, and flat frequency characteristics in the range of ~10 kHz was developed. From the results of a comparative analysis of the voltage sensitivity and frequency response characteristics of the developed KNN-based sensor and the commercial PZT-based sensor through an excitation test, the voltage sensitivity of the KNN sensor was found to have superior sensitivity with a smaller error than the commercial PZT-based sensor at the designed voltage sensitivity of 100 mV/g, and the resonant frequency and frequency flatness characteristics were almost equivalent. Furthermore, through a comparative evaluation of the detection performance of the two sensors for vibration generated during the operation of an actual pump facility, the stationary signal characteristics of the measured pump vibration signals of the two sensors were confirmed to be similar. This indicates that the vibration detection performance of the developed eco-friendly sensor is as excellent as that of the commercial PZT-based sensor. FFT analysis that converts the vibration signal data in the time domain to the frequency domain confirmed that both the KNN-based sensor and the commercial PZT-based sensor revealed peaks related to the pump speed and ball bearing defects at the same frequency location. From the above results, it was confirmed that the lead-free IEPE accelerometer developed in this study has performance and reliability equivalent to or superior to that of commercial PZT-based sensors. The findings of this study, therefore, demonstrate the possibility of the developed sensor replacing commercial PZT-based sensors in the condition monitoring and fault diagnosis of facilities and structures at various industrial sites.

## Figures and Tables

**Table 1:** Components and materials of the IEPE accelerometer.

Exploded Diagram	No.	Constituent Component	Material
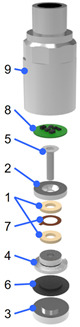	1	Piezoceramic rings	KNN-BNKLZ-0.01BS
	2	Head or seismic mass	Tungsten
	3	Base plate	Stainless steel
	4	Tail	Stainless steel
	5	Screw	Stainless steel
	6	Insulating layer	Epoxy
	7	Electrode	Inconel
	8	IEPE circuit	-
	9	Housing	Stainless steel

**Table 2:** Calculated frequency values of BSF, BPFO, BPFI, and FTF and the measurement results of the commercial sensor and KNN sensor.

Bearing No.	Bearing Position		Ball Spin Frequency (BSF)	Ball Pass Frequency Outer Race (BPFO)	Ball Pass Frequency Inner Race (BPFI)	Fundamental Train Frequency (FTF)
6320	Pump Drive End (DE)	Calculated	40.12 Hz	60.38 Hz	96.96 Hz	7.47 Hz
		Measured (Commercial sense)	40.00 Hz	62.00 Hz	96.50 Hz	-
		Measured (KNN sensor)	42.00 Hz	62.00 Hz	96.50 Hz	-
6226	Motor Drive End (DE)	Calculated	53.89 Hz	72.96 Hz	104.04 Hz	8.06 Hz
		Measured (Commercial sense)	51.50 Hz	71.50 Hz	104.00 Hz	-
		Measured (KNN sensor)	51.50 Hz	71.50 Hz	104.00 Hz	-

## Data Availability

Data are contained within the article.
